# Polymorphisms in *IL36G* gene are associated with plaque psoriasis

**DOI:** 10.1186/s12881-018-0742-2

**Published:** 2019-01-11

**Authors:** Tanel Traks, Maris Keermann, Ele Prans, Maire Karelson, Ulvi Loite, Gea Kõks, Helgi Silm, Sulev Kõks, Külli Kingo

**Affiliations:** 1 Department of Dermatology and Venerology, University of Tartu, 31 Raja St, 50417 Tartu, Estonia; 2 Clinic of Dermatology, Tartu University Hospital, 31 Raja St, 50417 Tartu, Estonia; 3 Department of Pathophysiology, University of Tartu, 19 Ravila St, 50411 Tartu, Estonia; 4 Perron Institute, Murdoch University, 8 Verdun St, Perth, WA 6009 Australia

**Keywords:** Plaque psoriasis, IL36G, Cytokines, SNP, Genetic association study

## Abstract

**Background:**

Plaque psoriasis is a non-contagious skin disease in which characteristic red and flaky lesions result from a dysregulation involving both innate and adaptive immune mechanisms. Several cytokines have been implicated in these processes and lately interleukin (IL)-36 family members have become more recognised among them. Thus far, genetic studies have only investigated *IL36RN* gene of this family in relation to pustular psoriasis. Since *IL36G* has previously demonstrated markedly increased levels in plaque psoriasis patients and is linked to IL-23/IL-17 axis critical in psoriasis pathology, it was chosen to be the focus of current report.

**Methods:**

Eleven SNPs from *IL36G* region were genotyped in 728 plaque psoriasis patients and 320 healthy control individuals. Allele and haplotype frequencies between patients and controls were assessed by respective association tests. For more specific analyses, the patients were assigned into subgroups according to sex, age of disease onset, occurrence of psoriasis among relatives, seasonal aggravation, arthritis symptoms, body surface area (BSA) scores, and Psoriasis Area and Severity Index (PASI) scores.

**Results:**

The most significant results were obtained with SNPs rs28947206, rs28947207 and rs28947211 that were associated in entire plaque psoriasis analysis (multiple testing adjusted *p* value (p_adj_) = 0.0054, p_adj_ = 0.0017 and p_adj_ = 0.0001) and also several subgroups. The first two of those SNPs were included in the same haplotype block with rs28947205 and rs12328178, and two of the respective haplotypes, CAGC and TGTT, provided similarly significant associations (p_adj_ = 0.0462 and p_adj_ = 0.0047).

**Conclusions:**

The associated SNPs of this study or those in linkage disequilibrium with them could potentially affect the functionality of IL-36γ cytokine, which in turn may impact plaque psoriasis pathology. For instance, these variants could influence IL-36γ expression or 3D structure, thereby altering its ability to induce chemokine production in keratinocytes and various immune cells. The precise mechanisms of these actions are currently unknown and out of the scope of this study. To conclude, the present genetic association results confirm the proposed role of IL-36γ in plaque psoriasis development, with corresponding causal effects to be determined in forthcoming research.

## Background

Psoriasis is a common non-infectious chronic skin disorder that is associated with considerable physical and social burden [1, 2]. Its estimated prevalence varies globally and is highest in western countries, where it affects around 2–4% of the population [3]. The most common form is plaque psoriasis, accounting 90% of all cases and manifesting as sharply demarcated erythematous plaques covered by silvery lamellar scales. Plaques can be few or extend over larger areas, and can also involve the entire body surface as erythroderma in extreme cases [4]. Concurrently, it has become evident that the effects of psoriasis are not exclusively confined to the skin, a notion supported by the regularly observed associations with different systemic diseases ranging from autoimmune to cardiovascular and psychiatric disorders [5].

Based on the pathological mechanisms that contribute to dermatological symptoms, psoriasis is considered an immune-mediated disease. The characteristic lesions develop through a complex interplay between both innate and adaptive immune system components [6]. Genetic research has underscored the crucial role of these interactions by identifying numerous susceptibility loci that contain immune-related genes, providing valuable insights into the pathogenic processes [7]. Central mechanisms can be divided into 1) the cytokine-mediated cross-talk between innate and adaptive immune systems involving tumor necrosis factor α (TNF-α), interferon γ (IFN-γ) and interleukin 1 (IL-1); 2) the IL-23/T helper cell 17 (Th17) axis; and 3) the effect of immune reactions on other skin cells [4].

More recently, pro-inflammatory IL-36 family cytokines have emerged as important drivers in psoriasis pathology [8]. All three members of the family, IL-36α, IL-36β and IL-36γ, are overexpressed in psoriasis lesional skin [9] and elevated IL-36α in a transgenic mouse model was shown to induce psoriasis-like symptoms [10]. Noticeably, the increased levels of IL-36α, IL-36β and IL-36γ in lesional skin were in good correlation with central cytokines of psoriasis pathology: IL-22, IL-17A, TNF-α and IFN-γ [11]. This suggests an inter-regulation that was further affirmed by intradermal injection of IL-36α into wild-type FVB mice, which lead to the increases of IL-36α itself, IL-17A, IL-23, TNF-α and IFN-γ mRNA [12]. On the other hand, injections of TNF-α, IL-17A, IL-23, IFN-γ, and IL-22 or their combinations induced IL-36α, IL-36β and IL-36γ [12]. All of this indicates that IL-36 cytokines synergize with prominent IL-23/Th17 axis in producing the psoriasis symptoms [13]. Additionally, cathelicidin (LL37) functions as an alarmin by responding to stimuli such as infection and wounding in the skin and displays aberrant expression in psoriasis, rosacea, and other inflammatory skin disorders [14–16]. Considering its proposed triggering role in psoriasis [17], it is interesting to note that LL37 increases IL-36γ protein expression and release from keratinocytes, that both LL37 and IL-36γ are coordinately abundant in psoriasis epidermis and IL-36γ in turn upregulates the production of chemokine (C-X-C motif) ligand 1 (CXCL1), CXCL8, CXCL10 and CCL20 in keratinocytes [18]. Finally, from the genetics standpoint, only *IL36RN* gene (encodes an antagonist IL-36Ra for common IL-36 family receptor - IL-36R) polymorphisms have been analyzed to date, yealding associations with pustular form of psoriasis [19–21].

In accordance with the findings described above, our plaque psoriasis whole transcriptome analysis detected an up-regulation of *IL36A*, *IL36G* and *IL36RN* gene expression in psoriasis patients [22]. This was further confirmed in a more detailed report, wherein *IL36G* and *IL36RN* demonstrated the most pronounced changes and IL-36γ was also shown to be markedly increased on a protein level [23]. To expand on the described observations, selected polymorphisms of the most promising candidate, *IL36G*, were genotyped in order to determine their possible associations with psoriasis susceptibility.

## Methods

### Study sample

The plaque psoriasis patients were recruited at the Department of Dermatology, University of Tartu, Estonia as described previously [24]. All were Caucasian, unrelated, living in Estonia and had a clear clinical diagnosis of plaque psoriasis (*n* = 728, age range 18–89 years). The control group included healthy unrelated Caucasians (*n* = 320, age range 18–71 years) without a personal or family history of psoriasis. They were enrolled from medical students, health care personnel and patients presenting at the dermatological outpatient clinic with mild expression of either facial teleangiectasis or skin tags. To conduct additional analyses, the patients were assigned into different subgroups according to specific subphenotypes. The early onset group consisted of cases where the symptoms appeared at or before 40 years of age (*n* = 551) and late onset when they appeared after 40 years of age (*n* = 177). Familial group included those that had psoriasis among relatives (*n* = 315) and the absence of it designated sporadic cases (*n* = 410). Three groups were formed according to the body surface area (BSA) scores: BSA ≤ 10 (*n* = 182), BSA 11–30 (*n* = 258) and BSA ≥ 31 (*n* = 285). Likewise, three groups were formed according to the Psoriasis Area Severity Index (PASI) scores: PASI ≤10 (*n* = 233), PASI 11–20 (*n* = 225) and PASI ≥21 (*n* = 265). Seasonal aggravation of symptoms was used to distinguish the spring-summer (*n* = 42) and fall-winter (*n* = 533) groups. Patients with inflammatory arthritis symptoms formed the PsA+ group (*n* = 152). Finally, male (*n* = 400) and female (*n* = 328) patients were analysed separately against their respective controls (*n* = 137, *n* = 157).

### Preparation of genomic DNA and analysis of genetic polymorphisms

**Fig. 1:**
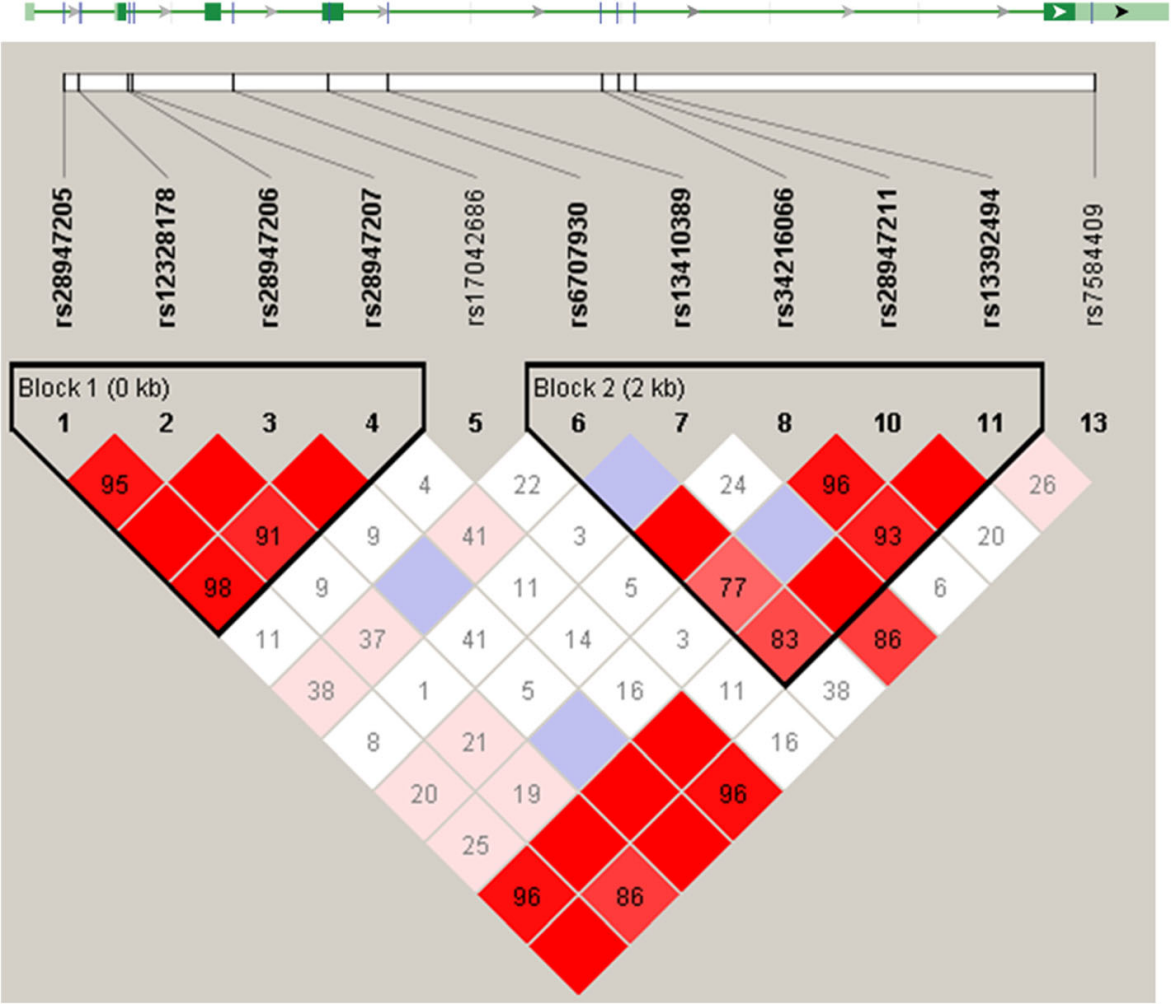
*IL36G* gene and genotyped SNPs. The image of LD pattern was generated using the Haploview v4.2 program and black boxes indicate haplotype blocks in entire psoriasis analysis

Genomic DNA was extracted from whole blood by standard high-salt extraction method and eleven single-nucleotide polymorphisms (SNPs) were genotyped using the OpenArray® Real-Time PCR Platform (Thermo Fisher Scientific, Waltham, Massachusetts, USA). The selected SNPs spanned almost the entire length of *IL36G* (Fig. 1).

### Data analysis

The Haploview v4.2 program was used for Hardy–Weinberg equilibrium (HWE) calculations in control group and subsequent allelic association and haplotype association tests between groups of patients and controls [25]. The Solid spine of LD algorithm (D’ > 0.8) integrated in Haploview v4.2 was applied to define the haplotype blocks and the resulting blocks were used in the haplotype association test. Differences in allele or haplotype frequencies between cases and controls were assessed by chi square test. The statistical significance threshold was set to 0.05 for all tests. The built-in permutation module of Haploview v4.2 was used to correct the *p* values for errors of multiple testing. Ten thousand permutations were performed, resulting in adjusted p values (p_adj_).

## Results

The genotyping procedure provided the genotypes of eleven studied SNPs (Table 1). All were polymorphic and met the inclusion criteria for Hardy-Weinberg equilibrium (Hardy-Weinberg p value > 0.05).

**Table 1:** Characteristics of studied SNPs

SNP	Position	pHW	MAF	Alleles (major/minor)	Feature
rs28947205	112,978,286	1.0	0.021	C/T	intron
rs12328178	112,978,391	1.0	0.013	A/G	intron
rs28947206	112,978,719	1.0	0.002	G/T	intron
rs28947207	112,978,748	1.0	0.011	C/T	intron
rs17042686	112,979,417	0.6071	0.066	G/C	intron
rs6707930	112,980,053	1.0	0.012	C/A	missense (Gln34Lys)
rs13410389	112,980,446	0.7871	0.085	G/A	intron
rs34216066	112,981,873	0.1303	0.095	T/C	intron
rs28947211	112,981,986	1.0	0.041	T/C	intron
rs13392494	112,982,098	0.175	0.464	C/T	intron
rs7584409	112,985,157	0.8851	0.31	A/G	3’ UTR

pHW – Hardy-Weinberg *p* value

MAF – minor allele frequency among healthy controls

3’ UTR – 3′ untranslated region

### Allelic association analysis

The strongest allelic associations were obtained with SNPs rs28947206, rs28947207 and rs28947211 (Tables 2 and 3). When analysing the entire psoriasis group, all three remained significant after correcting for multiple testing (p_adj_ = 0.0054, risk allele odds ratio (OR) 14.14, 95% confidence interval (CI) 1.93–103.81; p_adj_ = 0.0017, OR 4.43, CI 1.9–10.3; p_adj_ = 0.0001, OR 4.09, CI 2.04–8.17). In addition, rs28947206 associations withstood the correction in early onset (p_adj_ = 0.0145, OR 12.68, CI 1.7–94.48), late onset (p_adj_ = 0.0008, OR 18.66, CI 2.38–146.4), familial (p_adj_ = 0.003, OR 15.63, CI 2.06–118.7), sporadic (p_adj_ = 0.0122, OR 13.08, CI 1.73–98.96), fall-winter (p_adj_ = 0.0203, OR 11.98, CI 1.6–89.71), BSA ≤ 10 (p_adj_ = 0.0207, OR 12.7 CI 1.56–103.69), BSA 11–30 (p_adj_ = 0.0015, OR 16.98, CI 2.21–130.3), BSA ≥ 31 (p_adj_ = 0.0131, OR 12.7 CI 1.63–98.72), PASI ≤10 (p_adj_ = 0.0133, OR 12.88, CI 1.63–102.09), and PASI ≥21 (p_adj_ = 0.0003, OR 19.01, CI 2.5–144.46) group analyses. Rs28947207 demonstrated this in all the same groups and also male psoriasis analysis: early onset (p_adj_ = 0.0036, OR 4.17, CI 1.76–9.85), late onset (p_adj_ = 0.0009, OR 5.26, CI 2.05–13.47), familial (p_adj_ = 0.016, OR 3.83, CI 1.55–9.47), sporadic (p_adj_ = 0.0006, OR 4.94, CI 2.07–11.8), fall-winter (p_adj_ = 0.004, OR 4.15, CI 1.75–9.84), BSA ≤ 10 (p_adj_ = 0.0358, OR 3.84, CI 1.45–10.21), BSA 11–30 (p_adj_ = 0.0022, OR 4.62, CI 1.86–11.49), BSA ≥ 31 (p_adj_ = 0.0015, OR 4.67 CI 1.9–11.49), PASI ≤10 (p_adj_ = 0.0351, OR 3.69, CI 1.43–9.52), PASI ≥21 (p_adj_ = 0.0001, OR 5.97, CI 2.46–14.5), and male psoriasis (p_adj_ = 0.0191 OR 12.5, CI 1.7–91.64). Rs28947211 results remained significant in early onset (p_adj_ = 0.0001, OR 5.08, CI 2.24–11.48), familial (p_adj_ = 0.0019, OR 5.88 CI 2.01–17.18), sporadic (p_adj_ = 0.0201, OR 3.26 CI 1.49–7.14), fall-winter (p_adj_ = 0.0006, OR 4.33, CI 1.98–9.47), BSA ≤ 10 (p_adj_ = 0.0107, OR 13.21, CI 1.77–98.48), BSA ≥ 31 (p_adj_ = 0.0421, OR 3.45, CI 1.39–8.59), PASI ≤10 (p_adj_ = 0.0119, OR 5.62, CI 1.67–18.91), PASI 11–20 (p_adj_ = 0.0427, OR 4.18 CI 1.43–12.24), and female (p_adj_ = 0.0038, OR 5.51 CI 1.92–15.81) groups. The remaining SNPs did not produce significant results after multiple testing correction in the entire psoriasis group, but it did occur in certain subgroup analyses. Namely, rs28947205 remained significant in PASI ≥21 (p_adj_ = 0.0295, OR 2.73, CI 1.36–5.48), rs12328178 in BSA ≥ 31 (p_adj_ = 0.0483, OR 3.21, CI 1.35–7.62) and PASI ≥21 (p_adj_ = 0.0019, OR 4.25, CI 1.82–9.92), and rs34216066 (p_adj_ = 0.0031, OR 2.64, CI 1.52–4.59) in female psoriasis analysis. Additionally, SNPs rs6707930, rs13392494 and rs7584409 produced nominal associations in certain subgroups, but none of these withstood the correction.

**Table 2:** Results of allelic association analysis

SNP	Reference allele	Control RAF	Plaque psoriasis		Early onset		Late onset		Familial		Sporadic		Spring-summer		Fall-winter		PsA+		Male		Female	
			RAF	*P* value	RAF	*P* value	RAF	*P* value	RAF	*P* value	RAF	*P* value	RAF	*P* value	RAF	*P* value	RAF	*P* value	RAF	*P* value	RAF	*P* value
rs28947205	T	0.021	0.042	0.0197	0.039	0.0502	0.053	0.0086	0.036	0.112	0.047	0.0095	0.038	0.3414	0.040	0.037	0.035	0.2001	0.050	0.0417	0.033	0.5279
rs12328178	G	0.013	0.036	0.0064	0.035	0.0094	0.038	0.0124	0.034	0.0155	0.037	0.0071	0.038	0.094	0.035	0.0081	0.040	0.0098	0.042	0.0611	0.028	0.1299
rs28947206	T	0.002	0.023	**6.0E-4**	0.021	**0.0014**	0.030	**1.0E-4**	0.026	**4.0E-4**	0.021	**0.0013**	0.024	0.0036	0.020	**0.0021**	0.018	0.0069	0.026	0.0095	0.020	0.0548
rs28947207	T	0.011	0.045	**2.0E-4**	0.043	**4.0E-4**	0.053	**1.0E-4**	0.039	**0.0019**	0.050	**7.09E-5**	0.037	0.0594	0.042	**5.0E-4**	0.035	0.0137	0.049	**0.0015**	0.040	0.0945
rs17042686	C	0.066	0.076	0.4358	0.073	0.5793	0.085	0.2965	0.066	0.9968	0.083	0.2391	0.075	0.752	0.079	0.3278	0.101	0.0754	0.081	0.3216	0.069	0.9764
rs6707930	A	0.012	0.005	0.1014	0.003	0.0424	0.010	0.8038	0.007	0.4366	0.003	0.0577	0.000	0.3314	0.004	0.1099	0.007	0.5659	0.007	0.6401	0.002	0.013
rs13410389	A	0.085	0.071	0.3241	0.074	0.4436	0.064	0.3023	0.057	0.0852	0.084	0.9262	0.121	0.3308	0.071	0.3225	0.075	0.6474	0.063	0.0582	0.081	0.7473
rs34216066	C	0.095	0.064	0.0172	0.060	0.0104	0.075	0.3267	0.055	0.0114	0.071	0.1139	0.013	0.0148	0.062	0.0204	0.047	0.0171	0.080	0.238	0.044	**4.0E-4**
rs28947211	C	0.041	0.010	**1.75E-5**	0.008	**1.58E-5**	0.017	0.058	0.007	**3.0E-4**	0.013	**0.0018**	0.000	0.0627	0.010	**6.83E-5**	0.008	0.0095	0.012	0.0635	0.009	**4.0E-4**
rs13392494	T	0.464	0.515	0.0822	0.522	0.0568	0.492	0.4916	0.526	0.0668	0.507	0.1855	0.407	0.4282	0.511	0.1284	0.442	0.5997	0.540	0.1847	0.482	0.9231
rs7584409	G	0.310	0.328	0.4439	0.330	0.4223	0.323	0.7017	0.329	0.502	0.329	0.4687	0.300	0.8522	0.326	0.5148	0.324	0.6925	0.331	0.8454	0.326	0.6515

RAF – reference allele frequency

*P* values < 0.05 after 10,000 permutations are bolded

**Table 3:** Results of allelic association analysis

SNP	Reference allele	Control RAF	BSA ≤ 10		BSA 11–30		BSA ≥ 31		PASI ≤10		PASI 11–20		PASI ≥21	
			RAF	*P* value	RAF	*P* value	RAF	*P* value	RAF	*P* value	RAF	*P* value	RAF	*P* value
rs28947205	T	0.021	0.041	0.0799	0.045	0.0243	0.041	0.0504	0.036	0.1314	0.034	0.1804	0.054	**0.0032**
rs12328178	G	0.013	0.033	0.0344	0.033	0.0246	0.039	**0.0053**	0.031	0.045	0.022	0.2429	0.051	**3.0E-4**
rs28947206	T	0.002	0.021	**0.0023**	0.028	**2.0E-4**	0.021	**0.0018**	0.021	**0.0018**	0.017	0.0075	0.031	**7.38E-5**
rs28947207	T	0.011	0.039	**0.0038**	0.047	**3.0E-4**	0.048	**2.0E-4**	0.038	**0.0039**	0.036	0.0060	0.060	**8.64E-6**
rs17042686	C	0.066	0.098	0.0834	0.062	0.8463	0.073	0.6552	0.095	0.0982	0.071	0.7567	0.061	0.7902
rs6707930	A	0.012	0.000	0.0539	0.005	0.2288	0.008	0.5211	0.002	0.1131	0.002	0.1145	0.009	0.6308
rs13410389	A	0.085	0.056	0.1455	0.096	0.5856	0.058	0.1116	0.071	0.4649	0.083	0.9044	0.060	0.1548
rs34216066	C	0.095	0.059	0.0642	0.073	0.2135	0.059	0.0262	0.054	0.0175	0.084	0.5635	0.056	0.0193
rs28947211	C	0.041	0.003	**0.0011**	0.013	0.0093	0.012	**0.0047**	0.008	**0.0017**	0.010	**0.0047**	0.013	0.0087
rs13392494	T	0.464	0.508	0.2753	0.500	0.3266	0.534	0.0452	0.519	0.1425	0.471	0.8657	0.549	0.0159
rs7584409	G	0.310	0.308	0.9415	0.317	0.8073	0.348	0.1793	0.295	0.6002	0.303	0.8179	0.377	0.0239

RAF – reference allele frequency

*P* values < 0.05 after 10,000 permutations are bolded

### Haplotype analysis

The studied SNPs formed two haplotype blocks in the entire psoriasis analysis (Fig. 1). The first block consisted of rs28947205, rs12328178, rs28947206 and rs28947207 and the second block included rs6707930, rs13410389, rs34216066, rs28947211 and rs13392494. The first block had the same composition in all of the subgroups, whereas the second block had the last SNP rs13392494 omitted in early onset and BSA 11–30 analyses. In the case of familial, fall-winter, female and PASI 11–20 groups the second block was split into two, resulting in block 2 consisting of rs6707930 and rs13410389 and block 3 consisting of rs34216066, rs28947211 and rs13392494.

The most significant haplotype associations involved block 1 haplotypes CAGC and TGTT. Both of them withstood the correction for multiple testing in entire psoriasis analysis (p_adj_ = 0.0462, OR 0.45, CI 0.25–0.8, and p_adj_ = 0.0047, OR 8.08, CI 2.01–32.53; Table 4). Further, TGTT associations remained significant in early onset (p_adj_ = 0.0087, OR 7.8, CI 1.85–32.95), late onset (p_adj_ = 0.0018, OR 10.49, CI 2.23–49.26), familial (p_adj_ = 0.0031, OR 8.7, CI 2.07–36.49), sporadic (p_adj_ = 0.0096, OR 8.5, CI 1.84–39.3), fall-winter (p_adj_ = 0.0112, OR 7.6, CI 1.79–32.23), BSA 11–30 (p_adj_ = 0.0019, OR 9.54, CI 2.17–41.94), BSA ≥ 31 (p_adj_ = 0.0102, OR 8.55, CI 1.8–40.58), PASI ≤10 (p_adj_ = 0.0235, OR 7.96, CI 1.62–39.02), PASI ≥21 (p_adj_ = 0.0002, OR 11.46, CI 2.66–49.46) and male psoriasis (p_adj_ = 0.0544, OR 15.18, CI 1.17–196.9) analyses. CAGC associations remained significant in late onset (p_adj_ = 0.0142, OR 0.35, CI 0.18–0.68), sporadic (p_adj_ = 0.0183, OR 0.39, CI 0.21–0.72) and PASI ≥21 (p_adj_ = 0.0029, OR 0.34, CI 0.18–0.64) analyses. Block 2 haplotypes did not produce as strong associations in entire psoriasis group and the only result to survive permutation testing involved haplotype CGCC in early onset psoriasis (p_adj_ = 0.026, OR 0.32, CI 0.15–0.7). In the case of block 3, haplotype CCT withstood this correction in familial (p_adj_ = 0.0057, OR 0.3, CI 0.15–0.63), fall-winter (p_adj_ = 0.0019, OR 0.34, CI 0.19–0.62) and female (p_adj_ = 0.0014, OR 0.24, CI 0.11–0.52) psoriasis groups.

**Table 4:** Results of haplotype analysis in entire psoriasis group

Haplotype	Frequency	Controls	Patients	Chi Square	*P* value
Block 1					
CAGC	0.957	0.976	0.948	7.9	**0.0049**
TGTT	0.020	0.004	0.028	12.198	**5.0E-4**
TGGT	0.016	0.018	0.016	0.076	0.7831
Block 2					
CGTTC	0.493	0.509	0.486	0.88	0.3482
CGTTT	0.359	0.327	0.374	3.745	0.053
CATTT	0.080	0.083	0.078	0.109	0.7409
CGCTT	0.039	0.034	0.041	0.519	0.4714
CGCCT	0.015	0.011	0.024	4.493	0.034

*P* values < 0.05 after 10,000 permutations are bolded

## Discussion

Psoriasis develops in a multifactorial process influenced by the immune system, psoriasis-associated genetic susceptibility loci, autoantigens, and various environmental factors [26]. The possible triggers include mild trauma, sunburn, chemical irritants, systemic drugs, occupational risk factors impairing the skin barrier function, and HIV infection [4]. Estimated heritability at 66–90% is among the highest of all multifactorial genetic diseases, indicating the substantial impact of genetic susceptibility [27, 28]. The major risk loci that have been identified contain genes related to skin barrier functions, IL-23/Th17 axis, nuclear factor-κB and interferon signaling, and antigen presentation, thereby uncovering pathways behind the disease [27, 29]. Still, it is estmated that only about 25% of psoriasis heritability has been accounted for through all genetic discoveries published [30].

It is well established that pro-inlammatory cytokines play a prominent role in pathways leading to psoriasis. For instance, TNF-α released by both T cells and antigen-presenting cells facilitates the influx of inflammatory cells into lesional skin through induction of adhesion molecules, promotes the synthesis of other pro-inflammatory mediators, and activates dermal macrophages and dendritic cells [31]. Importantly, TNF-α also induces the production of another pivotal cytokine, IL-23, in myeloid dendritic cells [32]. This in turn causes the activation of Th17 cells and the effects of elevated IL-17, including epidermal hyperplasia, epidermal cell proliferation, and leukocyte infiltration into the skin [26]. Similarly to above cytokines, increased expression of IL-36α, IL-36β, IL-36γ and IL-36Ra has been detected in psoriasis patients [9, 23]. Notably, it has been demonstrated that IL-36 can be induced in human keratinocytes by TNF, IL-17, IL-22, and IL-36 itself; and conversely, IL-36 promotes TNF, IL-6 and anti-microbial peptides in keratinocytes [11]. It has been proposed, that the primary cellular effect of IL-36 cytokines in psoriasis is their impact on neutrophil inflammation through activation of neutrophil chemokines CXCL1 and CXCL8 [33, 34]. Genetic studies have also lend support for the relevance of IL-36 family and have thus far focused on *IL36RN*. Different mutations in this gene, that caused substantial impairments in IL-36Ra protein, were associated with generalized pustular psoriasis, a rare and severe form of the disease [19–21]. Because our preceding findings implicated IL-36γ as a primary suspect [22, 23], we decided to concentrate on its gene which is located 73 kb in 5′ direction from *IL36RN*.

The *IL36G* gene is located in the chromosomal region 2q14.1 together with the rest of IL-36 subfamily genes *IL36A*, *IL36B* and *IL36RN*. They all belong to the extended IL-1 family of cytokines and the genes of its five other members (*IL1A*, *IL1B*, *IL37*, *IL1F10/IL38* and *IL1RN*) surround the *IL36* cluster. The length of *IL36G* is 7653 bp, containing 5 exons. Eleven SNPs were selected from the gene for the herein presented genetic association analysis that provided a number of statistically significant results. The strongest among them concerned SNPs rs28947206, rs28947207 and rs28947211, which were associated in entire psoriasis analysis and also several subgroups. The first two SNPs were included in a haplotype block and its haplotypes CAGC and TGTT produced similarly significant associations. From the remaining SNPs, rs28947205, rs12328178 and rs34216066 were only associated in certain subgroup analyses. It should be noted, that many of the accompanying 95% confidence intervals were large, albeit did not cross the value of 1. This is due to the low frequencies of associated alleles and haplotypes and should be addressed in upcoming research by increasing the sample size.

To our knowledge, this is the first report describing *IL36G* variants in relation to psoriasis or any other condition. The physically closest SNP previously discussed is rs1374284 that was associated with therapy-related myeloid leukemia susceptibility and is located 10.3 kb from *IL36G* in 3′ direction [35]. Since none of the associated SNPs affect the peptide sequence of IL-36γ, they may exert regulatory control over *IL36G* expression or be in linkage disequilibrium with truly causal polymorphisms. In addition to transcription control, those causal variants could impact the conformation of IL-36γ protein, thereby influencing its ability to bind to IL-36R and induce the downstream signals. Since proteolytic processing of IL-36γ peptide is required to activate its proinflammatory activity [36, 37], respective cleavage sites may also be affected. However, as mentioned above, the *IL36G* gene has not been thoroughly studied and possible functional polymorphisms have not been determined. Therefore, to confirm the significance of the genetic associations presented here, these precise mechanisms would have to be uncovered in future research.

## Conclusions

Continuously accumulating evidence has suggested a role for IL-36γ in psoriasis pathology and the objective of this study was to investigate, whether the variants of its gene could potentially be associated with the disease. For this purpose, eleven SNPs from *IL36G* region were genotyped in 728 plaque psoriasis patients and 320 healthy control individuals. The following statistical analyses resulted in three significantly associated SNPs and two haplotypes, while the remaining associations were more modest. This indicates that *IL36G* polymorphisms could possibly affect IL-36γ functionality and thereby influence psoriasis susceptibility. The precise mechanisms are presently unknown and require respective experiments.

## Abbreviations

BSA: Body surface area
CI: 95% confidence interval
CXCL1: Chemokine (C-X-C motif) ligand 1
HWE: Hardy–Weinberg equilibrium
IFN-γ: Interferon γ
IL: Interleukin
LL37: Cathelicidin
OR: Risk allele odds ratio
p_adj_: Multiple testing adjusted *p* value
PASI: Psoriasis Area and Severity Index
SNP: Single-nucleotide polymorphism
Th17: T helper cell 17
TNF-α: Tumor necrosis factor α
